# Fit-for-purpose phosphorus management: do riparian buffers qualify in catchments with sandy soils?

**DOI:** 10.1007/s10661-013-3586-4

**Published:** 2014-01-07

**Authors:** David Weaver, Robert Summers

**Affiliations:** 1 Department of Agriculture and Food-Western Australia, 444 Albany Hwy, Albany, Western Australia 6330 Australia; 2 Department of Agriculture and Food-Western Australia, 120 South Western Highway, Waroona, Western Australia 6215 Australia

**Keywords:** Sediment, Riparian buffers, Soluble, Filterable reactive phosphorus, Leaching, Subsurface flow

## Abstract

Hillslope runoff and leaching studies, catchment-scale water quality measurements and P retention and release characteristics of stream bank and catchment soils were used to better understand reasons behind the reported ineffectiveness of riparian buffers for phosphorus (P) management in catchments with sandy soils from south-west Western Australia (WA). Catchment-scale water quality measurements of 60 % particulate P (PP) suggest that riparian buffers should improve water quality; however, runoff and leaching studies show 20 times more water and 2 to 3 orders of magnitude more P are transported through leaching than runoff processes. The ratio of filterable reactive P (FRP) to total P (TP) in surface runoff from the plots was 60 %, and when combined with leachate, 96 to 99 % of P lost from hillslopes was FRP, in contrast with 40 % measured as FRP at the large catchment scale. Measurements of the P retention and release characteristics of catchment soils (<2 mm) compared with stream bank soil (<2 mm) and the <75-μm fraction of stream bank soils suggest that catchment soils contain more P, are more P saturated and are significantly more likely to deliver FRP and TP in excess of water quality targets than stream bank soils. Stream bank soils are much more likely to retain P than contribute P to streams, and the in-stream mixing of FRP from the landscape with particulates from stream banks or stream beds is a potential mechanism to explain the change in P form from hillslopes (96 to 99 % FRP) to large catchments (40 % FRP). When considered in the context of previous work reporting that riparian buffers were ineffective for P management in this environment, these studies reinforce the notion that (1) riparian buffers are unlikely to provide fit-for-purpose P management in catchments with sandy soils, (2) most P delivered to streams in sandy soil catchments is FRP and travels via subsurface and leaching pathways and (3) large catchment-scale water quality measurements are not good indicators of hillslope P mobilisation and transport processes.

## Introduction

Phosphorus (P) and nitrogen (N) loss from landscapes to waterways has been identified as a key influence over the frequency and intensity of algal blooms (Sharpley et al. 1994; Daniel et al. 1994). To minimise nutrient loss and the threat of algal blooms, a range of nutrient management practices have been proposed, tested, modelled and implemented. These practices and tools include fertiliser management (timing, solubility, soil testing), effluent management (land disposal, artificial fertiliser substitution), soil amendment (increased P retention for sandy soils), perennial pastures and riparian buffers (fencing, stock exclusion, off-stream stock watering, stock and vehicle crossings; Keipert et al. 2008; Neville et al. 2008). Riparian buffers provide a multitude of functions such as stabilisation of channels, filtration of nutrients and sediment, and ecosystem services such as water purification by controlling pathogens and bacteria (Barling and Moore 1994). Whilst some studies suggest that riparian buffers can reduce P loss by 90 % (Line et al. 2000), others suggest that buffer strips may offer a temporary solution as sinks in some years and sources in others (Omernik et al. 1981). McKergow et al. (2003) showed no impact of riparian buffers on P delivery in Western Australia (WA) whilst Stutter et al. (2009) identified various biogeochemical mechanisms by which riparian buffers could contribute to increased P losses. Given that riparian buffers have been variously reported as contributing to P retention and P loss, it is important to understand the key factors and mechanisms contributing to the diversity of riparian buffer effectiveness in P management.

Hoffman et al. (2009) emphasised the need to identify and quantify the major hydrological pathways in order to understand the ability of riparian buffers to mitigate P transport. This is because the mechanisms that support P retention are likely to be different for systems dominated either by overland flow or subsurface flow. For overland flow, riparian buffer strips function to filter nutrients associated with sediment by physically filtering and trapping hillslope-derived particulate P (PP) in surface runoff. Riparian buffers can also reduce stream bank and bed erosion and hence reduce P delivery from the erosion of high P subsoils (Laubel et al. 2003). When subsurface flow pathways dominate, P sorption properties of soil and sediment and their hydrologic properties become more important. Hoffman et al. (2009) reported for systems dominated by overland flow that total P (TP) was reduced by 32–93 %, whereas dissolved reactive P (DRP) had a net release of 71 % up to a net retention of 95 %. Physical processes such as sedimentation of PP appears to account for the most P reduction by riparian buffers (up to 128 kg P ha^−1^ year^−1^), followed by biological processes through plant uptake that can temporarily retain up to 15 kg P ha^−1^ year^−1^, whilst the retention of DRP is often below 0.5 kg P ha^−1^ year^−1^ (Hoffman et al. 2009). Previous riparian studies in south-west WA (McKergow et al. 2003, 2006a, b) identified that subsurface transport pathways were an important factor in limiting the effectiveness of riparian buffers for P management. McKergow et al. (2003) also proposed that the presence of buffers may reduce suspended sediment (SS) concentrations in streams to such an extent that DRP concentrations can increase because of reduced P sorption potential that would otherwise be provided by the SS, prior to deposition and retention in the stream bed.

McDowell et al. (2004) cited numerous sedimentary, biotic, chemical, hydrological and physical processes that may alter the retention, release or transformation of P once it has entered a stream. Additionally, McDowell and Sharpley (2001) compared deposited stream bank and stream bed sediment P chemistry and noted that bed sediments were more likely to release P and stream bank sediment was more likely to retain P. Stone and Mudroch (1989) noted that P release and retention within streams was inversely related to particle size and P sorption capacity of suspended material, suggesting that hydrological processes controlling sediment particle size has implications for P delivery and fate in river systems. All of these factors and their interactions are likely to significantly alter water quality signatures such that they cannot always be interpreted as reflecting the process by which P was mobilised and transported from the landscape, although there are clearly instances where they are (van der Perk et al. 2007). These observations suggest that reliance on catchment-based water quality signatures that show high levels of PP as an indicator that P mobilisation and transport processes are dominated by surface erosion and runoff seems problematic. Further investigation was considered warranted based on evidence gained in WA.

For example, a coarse-scale national audit and modeling of catchments, rivers and estuaries in Australia (NLWRA 2002) indicated that for south-west WA, SS supply was on average 0.3 tonnes ha^−1^ year^−1^ (range 0.1–0.6 tonnes ha^−1^ year^−1^) and that 4 % (range 0.7–21 %), 70 % (range 52–83 %) and 26 % (range 14–45 %) of the SS was derived on average from hillslope, gully and bank erosion, respectively. NLWRA (2001) similarly reported for south-west WA that 3, 49, 14, 7 and 27 % of the P loss was from hillslope PP, gully erosion PP, bank erosion PP, point source DRP and runoff DRP, respectively. Based on this data, it seems unlikely in this region that SS and PP would be derived from surface erosion, yet riparian buffers are often implemented or proposed (Environmental Protection Authority 2008; Department of Water, WA 2010; Department of Water 2011) as a principal control for diffuse pollution, despite their reported limited effectiveness in this environment (McKergow et al. 2003, 2006a, b). Implementation often occurs without cognizance of the dominant hydrological pathways (Ruprecht and George 1993), P forms and transport pathways, or their cost effectiveness compared to other nutrient management practices (Weaver et al. 2005). In order for riparian buffers to meet site-specific requirements, scientists and managers need to be sure that the practice can provide “fit for purpose” P management benefits by considering local data sources and studies that (1) identify the dominant hydrological and contaminant pathways, (2) identify the nutrient forms being transported from hillslopes to buffers, (3) identify whether catchment-scale water quality signatures reflect landscape P delivery processes or may be subsequently modified by other in-stream processes and (4) consider whether the practice contributes to pollution swapping or has other adverse impacts (Stevens and Quinton 2008).

The objectives of this study based in south-west WA were to better understand the reasons behind the reported ineffectiveness of riparian buffers and to evaluate whether riparian buffers provide a fit-for-purpose solution for P management in catchments where sandy soils dominate. Data and findings from various studies in south-west WA were assembled to assist in understanding the role of riparian buffers to manage P in environments where sandy soils dominate. Dominant hydrological and P pathways and P forms delivered from hillslopes through different pathways were studied using runoff plots. Additionally, catchment water quality studies and P retention and release characteristics of stream bank and catchment soils were used to explore the potential for water quality signatures to reflect landscape P delivery processes. The findings are discussed in the context of other local studies that reported limited effectiveness of riparian buffers for P management (McKergow et al. 2003, 2006a, b), and conceptual models to support these findings are proposed.

## Materials and methods

### Catchment environment

**Fig. 1:**
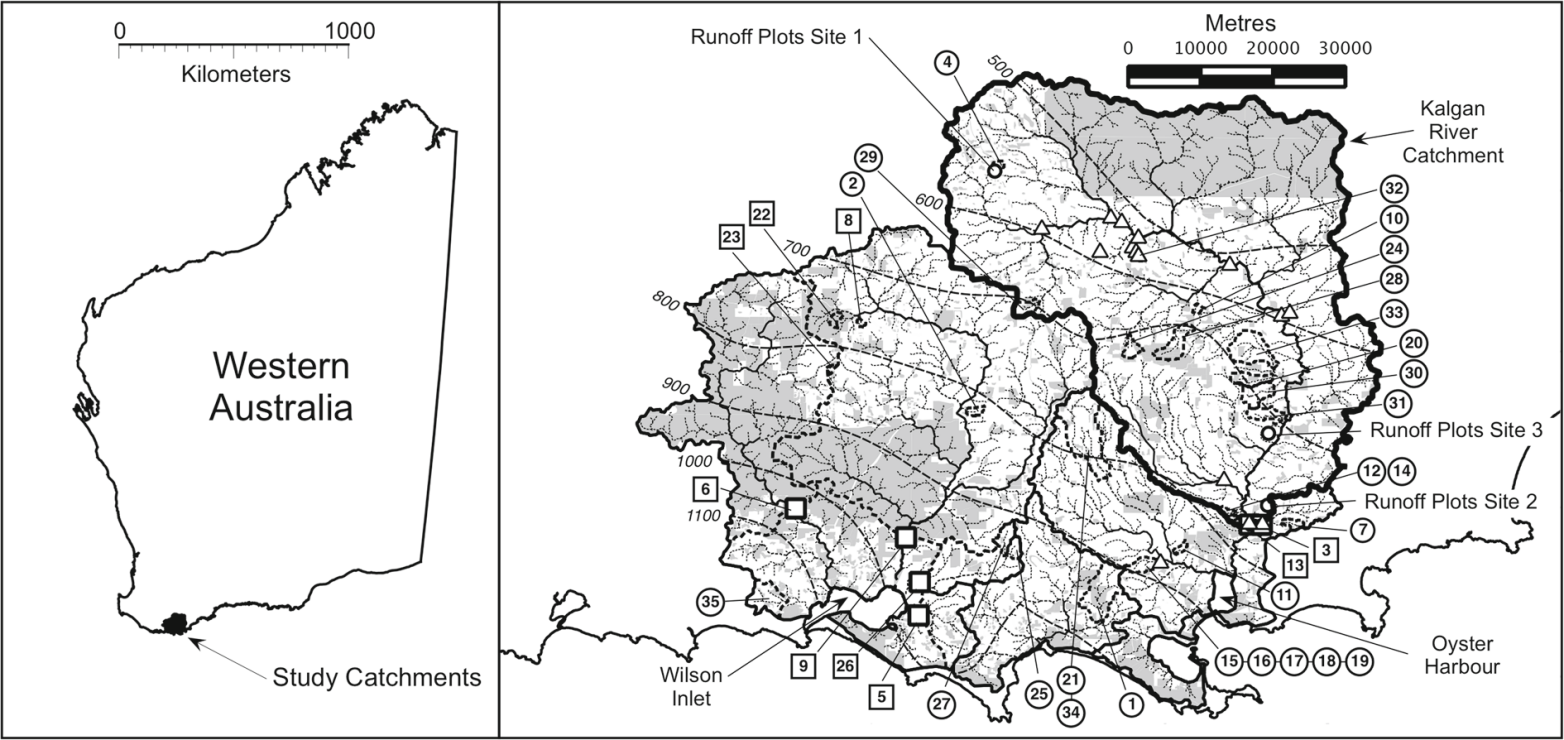
*Left panel*: Overview map showing the location of the study catchments in Western Australia. *Right panel*: Major catchment boundaries (*solid line*), Kalgan River catchment boundary (*thick solid line*), vegetation (*shaded*), stream network and rainfall isohyets (dashed *lines*). Runoff trial locations (*open circles*), monitored catchments (indicated by *numbered circles* and *dashed* catchment boundaries), location of stream bank sampling sites (*open triangles*) and Department of Water catchments (indicated by *numbered squares* and *white filled squares*). Refer to Table 1 to reconcile site numbers with summary water quality data

A Mediterranean climate, with cool wet winters and dry, temperate summers, typifies the region (Fig. 1). Annual average rainfall follows a strong gradient from 400 mm in the north-east of the region to 1,200 mm near the coast and dominates the winter months from April to October. The landscape comprises gently undulating plains developed mainly on tertiary sediments with occasional granitic hills (Churchward et al. 1988). Duplex soils are common with shallow grey acidic siliceous sands overlying laterite and clay at higher elevations and sands and sandy gravels at lower elevations, and valleys often comprise deep sands. The most common soil groups (duplex sandy gravel, grey deep sandy duplex and pale deep sandy soil) represent around 50 % of the study area (Schoknecht 2005). A land surface slope classification of the Oyster Harbour catchment on the south coast of WA (Fig. 1) shows that around 65 % has a slope of <4 % (Master 2007). Sixty percent of the study area was identified as having a potential for nutrient leaching, whilst only 15 % had a potential for surface soil erosion (Weaver et al. 2005). In the very flat catchment of the Peel-Harvey estuary on the west coast of WA, Ruprecht and George (1993) suggested that for deep sands, groundwater is permanently close to the surface (~1 m) and rises to provide overland flow in major storm events. Around 60 % of the P load for deep sands is transported by subsurface throughflow. For these duplex soils of the Peel-Harvey catchment, Ruprecht and George (1993) suggested that only around 25 % of the P load was transported by subsurface throughflow because of very low slopes and saturation of the sandy A horizon. The slope of the land surface in the south coast catchments under study here is greater than that of the Peel-Harvey, providing better drainage, resulting in more discharge via the B horizon subsurface pathways than via surface pathways or the A horizon (McKergow et al. 2006a). High subsurface flows only occur during winter, and high saturated conductivities (up to 18 m day^−1^) limit the residence time of water in subsurface soil layers. Piezometer and subsurface flow measurements suggest that subsurface flow contributes rapidly to streamflow during rainfall events, assisted by pockets of gravel, sharp soil horizon boundaries and macropores (McKergow et al. 2006a).

Around two-thirds of the landscape has been cleared of natural vegetation and replaced with agriculture, with most of the clearing taking place in the 1950s and 1960s. Around one-third of the landscape is used each for grazing, cereal cropping and natural vegetation (NLWRA 2001). Agriculture is typified by broadscale grazing of annual subterranean clovers (*Trifolium subterraneum* L.) and ryegrass for cattle and sheep in areas with higher rainfall (>600 mm annually), with the extent of cereal cropping increasing as rainfall decreases to <600 mm annually. Cereal cropping is supported by the use of minimum tillage. In their natural state, the soils are P deficient and will respond to applied fertiliser P. Fertiliser practice is typically based around tradition, with annual P applications of 10 − 15 kg P ha^−1^ year^−1^, independent of crop or pasture requirements (Weaver and Reed 1998). Many soils (63 % of pasture, 87 % of wheat, 89 % of dairy) now contain P in excess of requirements (Weaver and Wong 2011).

### Hillslope P transport

Runoff plots were established in three locations (upper, lower and bottom) in the Kalgan River catchment (the major tributary of Oyster Harbour, Fig. 1) on the south coast of WA in 1992 to assess the dominant hydrological and P pathways and to determine P leaching and runoff losses in relation to environmental conditions and P application over 2 years. The paddocks chosen had been used for agriculture, in particular the grazing of sheep or cattle on rain-fed annual pastures (ryegrass and subterranean clover), for around 30 years. At each location, uniform slopes within selected paddocks were identified and soil samples (consisting of 50 0–10-cm cores bulked) were analysed for bicarbonate-extractable P (Colwell P; Colwell 1965), ammonium oxalate extractable iron (Amox Fe; Tamm 1922) and aluminium (Amox Al), Phosphorus Retention Index (PRI; Bolland and Windsor 2007) and pH to assist in the interpretation of water quality data collected from the plots (Table 3). Phosphorus saturation ratio (PSR) was estimated for collected soils using the molar ratio method described by Chrysostome et al. (2007), and soil P status was determined using critical levels to achieve 95 % of maximum pasture production (Gourley et al. 2007). Six adjacent but hydrologically isolated plots (2 m wide and 40 m long) were established at each location. The plots were located midway down the slope and were hydrologically isolated by the use of a bund to ensure that there was no run on from the upper hillslope, and the use of 15-cm fibre cement sheeting strips was inserted to a 10-cm depth to ensure that there was no crossflow from adjacent plots. Surface runoff from each plot was directed into a covered 200-L drum, where depths were measured and converted into volumes based on a simple depth/volume calibration. Lysimeters, 30-cm diameter, were installed in the centre of each plot to determine volumes and quality of water leaching below 10 cm at each location. Three randomly selected plots at each location received the district average application of 10 kg P ha^−1^ in mid-June each year as superphosphate, whilst the other three plots received no P application. Water volumes were measured at varying intervals to match significant rainfall events and were converted to millimetres of rainfall equivalents for analysis. Subsamples of runoff and leachate waters were retained for analysis. Runoff samples were analysed for TP and a filtered subsample (<0.45 μm) for filterable reactive P (FRP), whilst leachate samples were analysed for FRP (APHA 1995). Water volumes and analyte concentrations were used to compute nutrient loads (kilograms per hectare) on an event and annual basis and aggregated according to specific analyses. Annual rainfall in each year of the trial was put into context with long-term annual rainfall at each site by comparing the rainfall against annual rainfall frequency distributions for data available since 1890 and 1975 (DSITIA 2013).

### Catchment P transport

Discharge measurements were coupled with TP, FRP and SS concentrations (Kisters Pty Ltd 2013) to derive annual TP and FRP loads for a wide range of catchments (Table 1) in the study area (Fig. 1) and TP, FRP and SS loads for the Kalgan River (Table 2) catchment (AWMA 1995), for varying periods from 1988 to 2000. The collection of gauging sites included small-scale sites established to estimate nutrient loss rates from various land uses and landscapes and longer-term sites that are part of the hydrological network managed by the Department of Water, WA (http://www.water.wa.gov.au). Instantaneous discharge (cubic metres per second) was determined from rating curves or discharge measurement structures established at gauging sites (Fig. 1) coupled with loggers and probes measuring stream water levels. Opportunistically collected grab samples of water were supplemented with samples collected by automatic samplers and point integrated air-displacement samplers on the rising stage (Guy and Norman 1970) in order to chemically characterise hydrographs for monitored catchments and subcatchments. Total P was determined on unfiltered samples using persulphate digestion (APHA 1995), and FRP was determined on filtered samples (<0.45 μm), both using the method of Murphy and Riley (1962). All unfiltered samples were also analysed for SS (APHA 1995; with a 1.2-μm GF/C filter paper). Where data was available, annual contaminant (TP, FRP, SS) loads were estimated by the integration of continuous discharge information with discontinuous time series chemistry. The contaminant load data were summarised by calculating the range and median with 95 % confidence interval and estimating the proportion of P delivered as FRP by dividing the median FRP load by the median TP load. Estimates of the unweighted proportion of P delivered as FRP were also made by calculating the mean and 95 % confidence interval of the ratios of measured FRP and TP concentrations at each site. The subcatchment- and catchment-scale data was compared to water quality data from runoff plots.

**Table 1:** Monitoring site numbers, catchment areas, median and range in TP and FRP loads, percentage of catchment and subcatchment P loads that is FRP, mean and 95 % confidence interval of FRP/TP concentration ratios and mean and 95 % confidence interval of unit area TP loads for sites in the study area

Number	Catchment area (ha)	Median load with range in parenthesis		FRP/TP load (%)	Median ± 95 % confidence interval	
		TP (kg)	FRP (kg)		Unit area TP load (kg ha^−1^)	FRP/TP concentration (%)
1	2,078	3,696 (1,560–4,353)	2,716 (1,194–3,055)	73	1.57 ± 0.63	70 ± 3
2	268	17 (6–49)	3 (1–6)	16	0.08 ± 0.08	37 ± 5
3	5,185	429 (137–3,055)	213 (181–1,528)	50	0.18 ± 0.12	63 ± 4
4	266	14 (4–26)	4 (1–5)	25	0.05 ± 0.03	27 ± 4
5	3594	1,782 (1,600–2,423)	568 (324–752)	32	0.54 ± 0.16	28 ± 5
6	64,127	930 (523–2,098)	247 (145–405)	27	0.02 ± 0.02	59 ± 13
7	331	52 (16–288)	24 (7–135)	47	0.28 ± 0.16	62 ± 5
8	105	5 (5–14)	0.6 (0.2–1)	11	0.08 ± 0.05	47 ± 4
9	121,100	1,481 (1,194–2,477)	458 (254–1,019)	31	0.01 ± 0.01	65 ± 11
10	150	nd	nd	nd	nd	39 ± 6
11	189	92 (52–197)	29 (13–147)	32	0.63 ± 0.39	52 ± 4
12	172	20 (11–64)	nd	nd	0.17 ± 0.11	79 ± 4
13	244,571	5,853 (316–41,534)	1,460 (574–3,689)	25	0.05 ± 0.02	52 ± 3
14	127	9 (4–33)	nd	nd	0.09 ± 0.02	84 ± 0
15	183	9 (6–18)	2 (2–10)	26	0.06 ± 0.03	40 ± 4
16	267	40 (17–74)	26 (3–61)	66	0.16 ± 0.11	60 ± 4
17	294	30 (20–71)	13 (5–48)	44	0.13 ± 0.08	52 ± 5
18	589	371 (112–793)	181 (77–525)	49	0.72 ± 0.4	65 ± 2
19	649	122 (70–274)	89 (54–222)	73	0.23 ± 0.14	75 ± 2
20	980	nd	nd	nd	nd	18 ± 4
21	3,318	1,030 (1,030–1,030)	992 (992–992)	96	0.31 ± 0	75 ± 8
22	137	3 (2–5)	0.6 (0.6–0.7)	20	0.03 ± 0.02	50 ± 15
23	115	3 (2–5)	0.4 (0.1–0.8)	14	0.03 ± 0.02	32 ± 3
24	95	33 (5–107)	6 (3–54)	18	0.41 ± 0.31	58 ± 11
25	669	130 (18–543)	105 (76–133)	80	0.31 ± 0.36	80 ± 7
26	7,592	3,615 (2,973–7,289)	2,018 (1,792–3,915)	56	0.61 ± 0.39	50 ± 4
27	624	41 (4–1,859)	33 (12–55)	82	0.78 ± 1.2	69 ± 13
28	1,190	nd	nd	nd	nd	21 ± 3
29	563	17 (17–17)	17 (17–17)	99	0.03 ± 0	68 ± 9
30	1,326	nd	nd	nd	nd	39 ± 8
31	559	43 (1–178)	1.6 (0.5–76)	4	0.12 ± 0.14	74 ± 5
32	224	6 (0–114)	0.9 (0.4–6.6)	16	0.13 ± 0.12	49 ± 15
33	2,098	26 (17–68)	17 (6–29)	64	0.02 ± 0.02	45 ± 4
34	3,664	740 (582–1,172)	513 (391–1,084)	69	0.23 ± 0.11	67 ± 6
35	1,718	nd	nd	nd	0.13 ± 0.04	41 ± 20
Runoff 1	0.008	0.0024 (0.0005–0.0549)	0.0023 (0.0004–0.0548)	96	2.70 ± 2.58	
Runoff 2	0.008	0.0552 (0.0213–0.0704)	0.0548 (0.0210–0.0701)	99	6.40 ± 1.00	
Runoff 3	0.008	0.0061 (0.0030–0.0103)	0.0059 (0.0030–0.0103)	97	0.81 ± 0.12	

*nd* not determined due to lack of discharge data or associated water chemistry

**Table 2:** Annual discharge summary, and discharge characteristics, annual TP, FRP, and FRP/TP ratio and SS loads, for the Kalgan River

Year	Flow (gigalitres)	Number of flow events^a^	Days where mean daily flow^b^ >10 m^3^ s^−1^	Maximum annual flow rate (m^3^ s^−1^)	TP load (tonnes)	FRP load (tonnes)	FRP/TP load (%)	SS load (tonnes)
1987	10	0	0	2.1	0.3	0.2^c^	51	29^d^
1988	113	9	31	117.8	42.8	6.5^c^	15	10,294^d^
1989	25	0	0	6.8	1.0	0.4^c^	41	162^d^
1990	46	1	7	59.7	8.9	3.3^c^	37	3,827^d^
1991	64	2	14	130.6	32.0	7.2^c^	23	11,962^d^
1992	75	4	22	60.7	9.6	2.8	29	4,550
1993	94	8	25	54.1	11.7	3.7	32	2,840
1994	37	2	5	21.6	2.5	1.5	60	740
1995	25	0	0	10.1	0.9	0.6	67	280
1996	41	3	4	18.5	1.5	0.7	47	850

^a^Events characterised by maintenance of mean daily flow rate above 10 m^3^ s^−1^ for one or more consecutive days

^b^Total number of days where the mean daily flow rate exceeded 10 m^3^ s^−1^

^c^Estimated from a relationship between FRP and maximum annual flow rate for 1992–1996. FRP = 0.038 + 0.0552 × MaxQ. *R* ^2^ = 0.86

^d^Estimated from a relationship between SS and maximum annual flow rate for 1992–1996. log(SS) = 0.997 + 1.456 × log(MaxQ). *R* ^2^ = 0.98

### P characteristics of catchment and stream bank soils

Stream banks were sampled at 17 locations in the Oyster Harbour catchment to determine P content and P sorption characteristics in comparison to surface agricultural soils. Sites selected included currently eroding stream banks (Fig. 1, some sites overlap at the presented map scale). This was done to distinguish whether these materials would act as a P source or as a P sink for P lost through other pathways. Previous work in Australia had indicated that gully erosion could be a major P source contributing to downstream waterway pollution in comparison to other sources (Caitcheon et al. 1995). At each location, subsamples from eroding banks were combined to obtain a representative composite sample. Each stream bank sample was dried at 40 °C and fractionated into >1-, 0.6–1-, 0.3–0.6-, 0.15–0.3-, 0.075–0.15- and <0.075-mm-sized fractions. The fractions were analysed for total P after Kjeldahl digestion, Amox Fe and Amox Al (Tamm 1922), PRI (Bolland and Windsor 2007), bicarbonate-extractable P (Colwell P; Colwell 1965) and organic carbon (Walkley and Black 1934). Phosphorus saturation ratio (Chrysostome et al. 2007), Colwell P/PRI ratio and estimated FRP (Moody 2011) and TP (Dougherty et al. 2011b) concentrations likely to arise from soil or stream bank materials were derived. For all analytes or their derivatives, fractionated values were combined on a weight basis to derive analysis for whole stream bank materials. The whole stream bank data and the <75-μm fraction were compared with 422 surface (0–10 cm) samples of soil that had been collected in the study catchments. Soil samples underwent the same chemical analysis, but on bulk soil sieved to <2 mm.

### Data analysis

Summary statistics such as means, medians, ranges, 95 % confidence intervals and correlation and regression equations and statistics were calculated using DataDesk (Data Description 1996) and by the application of standard statistical methods described by Helsel and Hirsch (1992). Graphics, box and whisker plots, distribution plots, percentiles and related statistics were prepared using Aabel (Gigawiz Ltd. Co. 2013) and EazyDraw (Dekorra Optics LLC 2013), and maps were prepared using Quantum GIS (QGIS Development Team 2013). Flow and load data were derived using Hydstra/TS (Kisters Pty Ltd 2013).

## Results and discussion

### Hillslope P transport

**Fig. 2:**
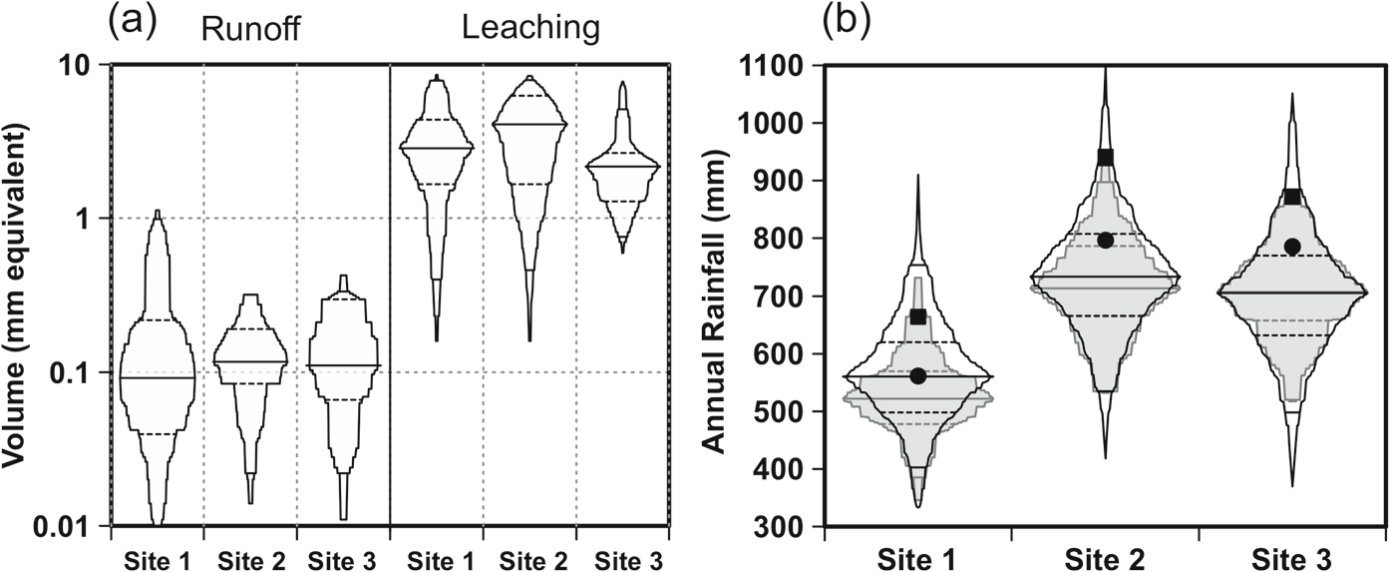
**a** Distribution plots showing volumes (millimetre equivalent) delivered via runoff or leaching vectors for sampling events at each runoff trial site and **b** distribution plots of long-term annual rainfall since 1890 at each site (*unfilled*), distribution plots of annual rainfall since 1975 at each site (*filled grey*), overlayed with annual rainfall in year 1 (*filled square*) and year 2 (*filled circle*) of the trials. Distribution plots range from minimum to maximum, with *dashed horizontal lines* showing 5th, 25th, 50th, 75th and 95th percentiles

Across all events, sites and treatments, significantly more water was leached than was delivered as surface runoff. Twenty-three times as much water was collected as leachate than runoff (Fig. 2a). This suggests that in this region for these experimental conditions, significantly more water travels vertically, rather than across the soil surface. The dominance of leaching as a transport vector is further reinforced in Fig. 2b which shows the annual rainfall at each site for each year of the trial in the context of distributions of long-term rainfall since 1890 and reduced rainfall since 1975 (IOCI 2010). For rainfall since 1890, the annual rainfall during the trial years was >50th percentile for site 1 and approximately 75th percentile for sites 2 and 3. For rainfall since 1975, annual rainfall at each site was >90th percentile in year 1 and approximately 75th percentile in year 2. Hence, whilst the years of the trials represented years where the likelihood of runoff was high, leaching was still the dominant process (Fig. 2a). This is consistent with the sandy texture of the soils, indicating a high winter rainfall infiltration. It is possible that rainfall may re-emerge further down the hillslope; however, leaching appears to be the dominant process in these sandy soils in these landscapes at the local scale. Nutrient transport losses at the hillslope scale are therefore more likely to be dominated by FRP than PP, influencing nutrient management practice performance.

**Fig. 3:**
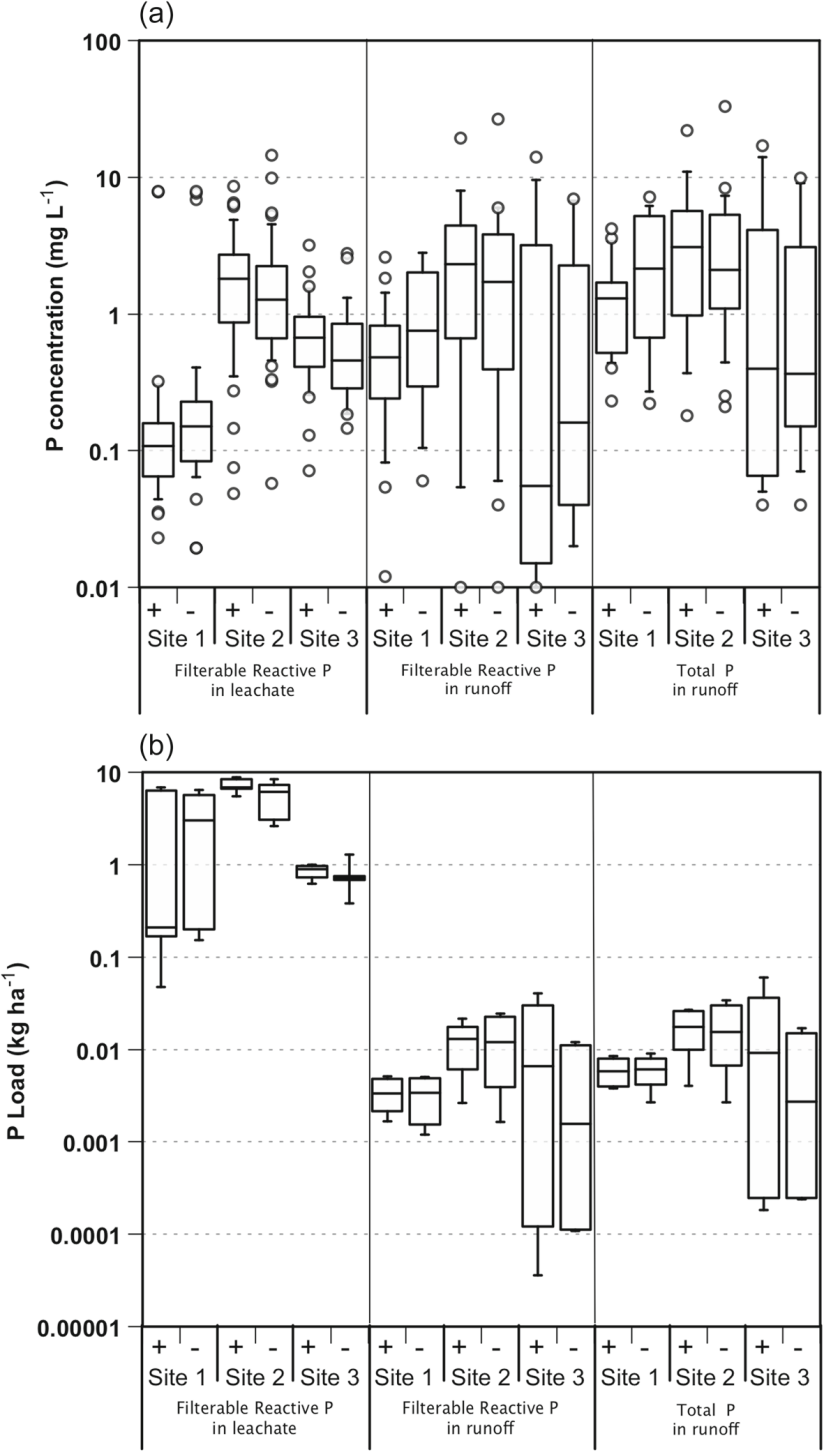
Box and whisker plots of **a** P concentrations (milligrams per litre) and **b** loads (kilograms per hectare) of FRP and TP for samples collected as leachate or runoff at each site over the 2-year monitoring period with (+) or without (−) P added. *Whiskers* show 10th and 90th percentiles, and *boxes* show 25th, 50th and 75th percentiles. *Circles* show outliers

Despite water yield data being consistent across all sites, there were some site and hydrological vector-specific differences in runoff or leachate P concentrations (Fig. 3a, b) that can be partly explained by different soil characteristics (Table 3). For example, site 2 showed no significant difference between leaching and runoff FRP concentrations. This may be due to higher PSR, suggesting that the top 10 cm of soil may be uniformly saturated with P, and hence, rainfall interaction with the surface layers will make little difference in FRP concentrations. In contrast, site 1 showed significantly higher FRP concentrations in surface runoff than in leachate. This site has the lowest PSR and hence is most likely to show strong stratification in soil P storage and P sorption with depth (Weaver et al. 1994; Dougherty et al. 2006), with low PSR arising because of dilution from very low PSR soil below the surface and higher PSR soil in the top few centimetres. Rainfall generating surface runoff would interact with high PSR soil in the top few centimetres, whilst rainfall generating leachate would interact overall with a lower PSR soil. Surface runoff would therefore result in FRP concentrations greater than those found in leachate. Sites 2 and 3 had higher PSR values than site 1 and generally showed higher FRP concentrations in leachate than site 1. That is, higher FRP concentrations were generally found in leachates with higher PSR values.

**Table 3:** Soil chemical and physical characteristics of the 0–10-cm layer at each runoff trial site

Parameter	Site 1	Site 2	Site 3
Texture (surface soil)	Sandy loam	Sandy loam	Sandy loam
pH (CaCl_2_)	5.1	4.8	4.4
Amox Al (mg kg^−1^)	2,600	180	110
Amox Fe (mg kg^−1^)	440	240	100
Colwell P (mg kg^−1^)	54	32	13
Total P (mg kg^−1^)	460	180	78
PRI (mL g^−1^)	64	−1.1	−0.9
PSR	1.7	7.1	9.4
Phosphorus status	High	High	High
Slope (%)	0.6	1.9	1.9

**Fig. 4:**
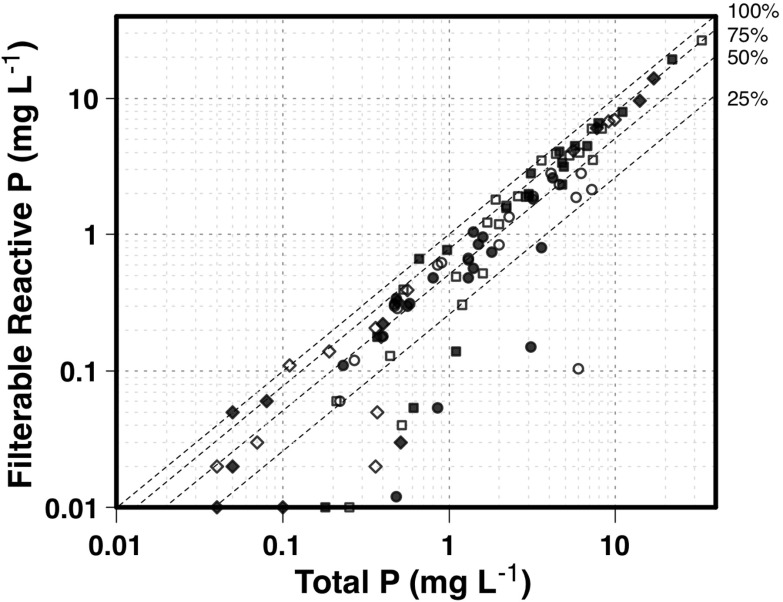
Filterable reactive P concentration (milligrams per litre) in surface runoff for site 1 (*circles*), site 2 (*squares*) and site 3 (*diamonds*) as a function of total P concentration (milligrams per litre) per sampled runoff event for plots with (*open symbols*) or without (*closed symbols*) P added. *Dashed isolines* show specified proportions of runoff samples as FRP

There were small differences between FRP and TP concentrations in surface runoff, indicating that a significant proportion of surface runoff P was as FRP. Independent of site and treatment, the median ratio of FRP to TP was 60 % (Fig. 4). Based on mean values of TP and FRP in surface runoff at site 1, 42 % of the P was FRP compared to 75 % at site 2 and 73 % at site 3. Differences between FRP and TP in surface runoff observed at site 1 are likely to be due to higher P sorption characteristics (Amox Fe, Amox Al, PRI), TP and Colwell P (Table 3). Discharge of particulates and PP in surface runoff at site 1 would contribute to TP, explaining the largest difference observed between FRP and TP of all of the sites. As site 1 contains the most soil P, particulate matter lost from the surface at site 1 is likely to discharge more PP than those indicated by the values in Table 3 because of stratification (Weaver et al. 1994; Dougherty et al. 2006) and enrichment due to selective loss of finer particulates during erosion (Quinton et al. 2001). However, particulate transport at site 1 is likely to be moderated by the low slope (Table 3). Hence, P mobilised in surface runoff from site 1 may contain FRP due to high soil P and P desorption from P-saturated surface soil; however, particulate matter contributing to TP may also resorb some of the mobilised FRP. At site 1 therefore, the difference between FRP and TP may not be as great as expected because of limited delivery of particulate matter due to low slopes. Figures 3a, b and 4 collectively indicate that FRP fractions are a major component of the P forms in both surface runoff and leachate, except where these can be moderated by passing through high P-fixing soil materials (site 1) or where fine enriched particulate material can both contribute to TP and moderate FRP concurrently.

Overall, there were no significant influences of the addition of P on FRP or TP concentrations compared to where no P was added (Figs. 3a and 4). A separation between the closed and open symbols in Fig. 4 would be expected if there was an influence of fertiliser P addition on P concentrations. This is not evident for reasons including (1) the lack of coincidence between fertiliser addition and significant rainfall events (Dougherty et al. 2011a), resulting in incorporation of applied P into the agricultural soils and plants, and (2) the fertiliser P additions (<10 mg P kg^−1^) are small compared to the P stored (78–460 mg P kg^−1^) in these fertile agricultural soils (Table 3).

**Fig. 5:**
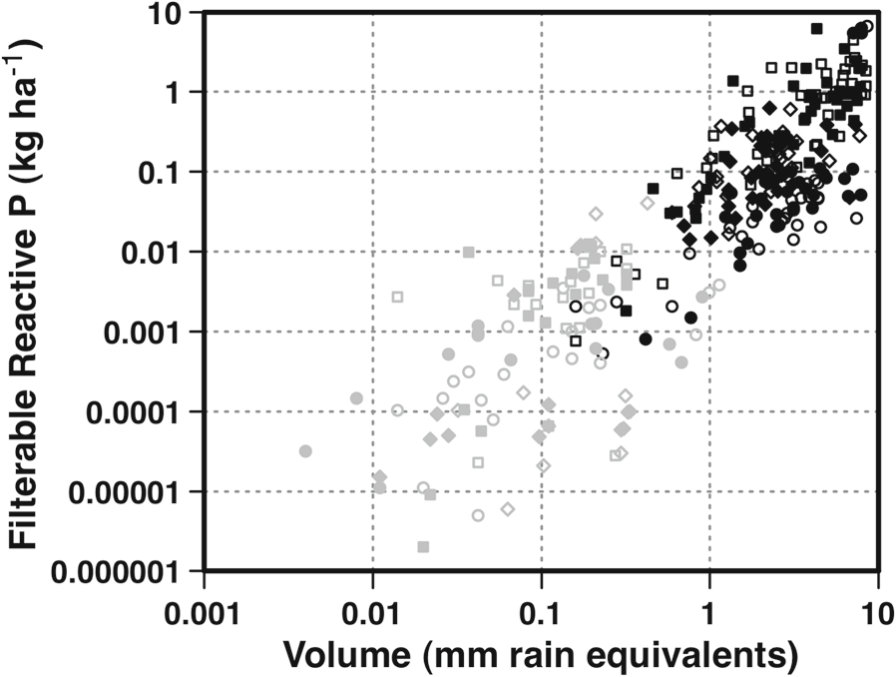
Filterable reactive P loads (kilograms per hectare) for site 1 (*circles*), site 2 (*squares*) and site 3 (*diamonds*) as a function of volumes discharged per event (millimetres of rainfall equivalents) as runoff (*grey symbols*) or leachate (*black symbols*) for plots with (*open symbols*) or without (*closed symbols*) P added

Given that there are major differences in the volumes of leachate and runoff at each site (Fig. 2), and little difference in FRP concentrations in leachate and runoff (Fig. 3a), the loads of FRP lost via leaching and runoff vectors were driven strongly by volume. The variation and magnitude of FRP and TP loads for each plot in each year delivered via different pathways shows that FRP loads in leachate were on average 2 orders of magnitude higher than FRP or TP in runoff (Fig. 3a, b). This is largely a function of greater volumes delivered via leaching pathways. This is further shown in Fig. 5 where there is a clear separation of FRP load for leachate and runoff events, with leachate providing significantly greater volumes and loads. It follows that these systems under the measured conditions are predisposed to deliver largely FRP via leaching. Overall, the high proportion of P lost as FRP via leaching pathways and small amounts of PP transported via runoff pathways translates into a 96 to 99 % of P lost as FRP via all measured pathways (Table 1).

Losses via surface runoff are also dominated by FRP as shown by the similarity between FRP and TP loads in surface runoff, but these are moderated where P retention capacity remains in the soil. Over the life of the experiment, site 1 delivered 56 % of its P load in surface runoff as FRP whilst sites 2 and 3 delivered 75 %. This has implications for riparian buffers since most P is transported as FRP, and most likely via subsurface transport pathways (McKergow et al. 2006a, b). The physical filtering opportunities provided by riparian buffers would therefore be bypassed, and even where surface runoff was occasionally a more dominant process (site 1), significant amounts of the transported P were in a FRP form (Figs. 3a and 4) which would not be filtered by buffers. Riparian buffers in this environment will therefore do little to filter FRP delivered via surface runoff (Fig. 4) and will also be unable to moderate most of the P transported via leaching (Figs. 3b and 5).

### Catchment P transport

The median P load increased with increasing catchment size (Table 1) for both FRP (log FRP load = 0.689 × log area − 0.461, *R* ^2^ = 0.67) and TP (log TP load = 0.737 × log area − 0.146, *R* ^2^ = 0.75), and similar relationships have been reported elsewhere (Prairie and Kalff 1986). There was a considerable variation in annual P loads, which translated into a wide range for the 95 % confidence intervals for the median values of TP and FRP. The FRP/TP load ratio (an indicator of the percentage of soluble P) varied across catchments and ranged from 4 to 99 %, with a mean value of 45 %. A regression of median FRP load as a function of median TP load (not shown) suggests that 41 % of the P load is FRP and is consistent with the large-scale modelled estimates of 34 % from NLWRA (2001). This implies that around 40 % of the P loads in this study area for catchments from 100–245,000 ha are measured as FRP, and the other 60 % are therefore PP. The larger catchments (sites 6, 9 and 13) all had similar proportions (27, 31 and 25, respectively) of the measured P load as FRP, whilst the remaining smaller catchments (<7,500 ha) ranged from 4 to 99 % of the P load as FRP. Site 29 and site 1 had 99 and 73 % of the P load as FRP, respectively, and were known to be influenced by primary treated sewerage and discharge from a piggery. This partially explains the high FRP percentage, whilst most other catchments are influenced predominantly by diffuse agricultural nutrient sources. Table 1 also shows the mean and 95 % confidence interval of FRP/TP concentration ratios. In many cases, these FRP/TP concentration ratios are greater than the equivalent FRP/TP load ratio, and across all subcatchments and catchments, the mean FRP/TP concentration ratio was 57 %. The lower FRP/TP load ratio is to be expected since load calculations account for variations in both volume and concentration. Changes in volume and concurrent changes in P concentrations and the ratio of FRP/TP will lead to FRP/TP load ratios that are different to FRP/TP concentration ratios. For example, higher flow or poor management usually increases suspended material that would decrease the FRP/TP concentration ratio and, overall, reduce the FRP/TP load ratio (McKergow et al. 2003). This is evident in Table 2 for the Kalgan River where flow is positively correlated with SS loads (Spearman's rank correlation = 0.784, *P* < 0.01) and TP loads (Spearman's rank correlation = 0.948, *P* < 0.001) and negatively with FRP/TP load ratios (Spearman's rank correlation = −0.845, *P* < 0.01). Compared to years of higher flow showing FRP/TP load ratios of approximately 30 %, years of lower flow (1994–1996) show higher FRP/TP load ratios (47–67 %), fewer flow events of shorter duration and lower maximum annual flow rates. The years of higher flow had more flow events of longer duration and higher maximum annual flow rates.

Catchment-scale data (Tables 1 and 2) is sometimes misinterpreted to conclude that P is mobilised and transported from hillslopes to streams in a PP form (that is not leached and with low FRP/TP ratios), commensurate with water quality measurements observed at the large catchment scale. However, in-stream water quality values and P forms can be further influenced by in-stream factors (McDowell et al. 2004), and hence, without hillslope scale data, it is possible to erroneously conclude that water quality measurements at the catchment scale reflect nutrient mobilisation processes in operation at the hillslope scale. For these study catchments, using the large catchment-scale data alone, we may erroneously conclude that surface runoff and erosion is the dominant hillslope mobilisation process for P delivery since measurements showed 40 % FRP on average; however, the runoff plots had 96 to 99 % of the P lost as FRP. Using the catchment-scale data alone could lead to the incorrect conclusion and that riparian buffers would reduce P loss from the study catchments. In contrast to the commonly reported model of P mobilisation and transport by surface runoff and erosion, the data presented here may be interpreted by alternative models as follows: (1) P transport vectors differ in years dominated by different hydrological processes. Years where runoff is dominant would result in high PP loads and low FRP/TP load ratios, whilst years where leaching is dominant would lead to low PP loads and higher FRP/TP load ratios since leaching processes deliver mostly FRP (Table 2), and (2) stream sediment contributes, either through remobilisation and/or bank erosion, to increased SS loads and reduced FRP/TP load and concentration ratios in years of high flow because of in-stream P sorption. This alternative explanation may also be coupled with the notion that FRP lost from the landscape combines with stream sediment to provide varying FRP/TP signatures (Table 1, “P characteristics of catchment and stream bank soils” of the “Results and discussion” section). Each of these alternatives seems plausible in addition to the standard model of erosion of particulates from the landscape surface as the dominant process of P delivery and cause of water quality signatures measured in streams.

A comparison of the data collected from the runoff plots and the catchment-scale water quality measurements suggests a change in measures of P form from 96 to 99 % FRP at the hillslope scale to on average 40 % FRP at the large catchment scale (Table 1). This comparison suggests an influence of the stream network on P form and implies that measures of P form at the large catchment scale are not always indicators of nutrient loss mechanisms or of the potential success of management actions (Haggard and Sharpley 2007). The data also suggests that FRP lost from the landscape combines with in-stream sediment sources to provide the water quality signatures of 40 % FRP and 60 % PP at the large catchment scale. This is consistent with NLWRA (2001, 2002), which suggests that only 3 % of P is lost as hillslope PP and that 96 % of SS is derived from gully and bank erosion. This is further supported by the relationship between TP load and catchment size in this study (log TP load = 0.737 × log area − 0.146, *R* ^2^ = 0.75), where a slope less than unity leads to decreasing losses of P per unit area as catchment size increases. At the smallest scale, runoff plots (0.008 ha) measured an average loss of 3.3 kg P ha^−1^, catchments from 100–3,500 ha measured an average loss of 0.35 kg P ha^−1^, catchments from 3,500 − 64,000 ha measured an average loss of 0.13 kg P ha^−1^ and catchments greater than 64,000 ha measured an average loss of 0.04 kg P ha^−1^. These systematic decreases in unit area P load with increasing catchment size are unlikely to be due to dilution alone, and therefore, P lost from the landscape principally as FRP will be converted to PP within the stream and be subsequently assimilated and/or remobilised within the stream network through sedimentation and other processes (Prairie and Kalff 1986; Haggard and Sharpley 2007). This is consistent with NLWRA (2001) for south-west WA and Keipert et al. (2008) and Rivers et al. (2013) for the Peel Harvey catchment, who suggested that 39 and 20 % respectively of the mobilised P is delivered to catchment outlets, with the remainder retained in the hydrologic network.

### P characteristics of catchment and stream bank soils

**Fig. 6:**
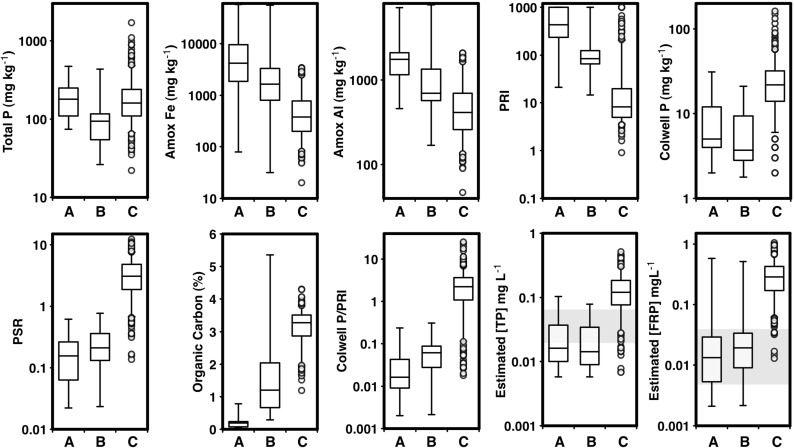
Box and whisker plots comparing chemical characteristics of (*A*) <75-μm fraction of stream bank soil, (*B*) whole stream bank soil and (*C*) <2-mm fraction of catchment soils. *Whiskers* show 5th and 95th percentiles, and *boxes* show 25th, 50th and 75th percentiles. *Circles* show outliers. *Shaded areas* show ANZECC/ARMCANZ water quality targets for south-west WA

Catchment soils have more TP and Colwell P than whole stream bank soil (Fig. 6). The <75-μm fraction of stream bank soil had more TP and Colwell P than the whole stream bank soils and around the same TP and less Colwell P than catchment soils. Increasing TP with decreasing particle size has been reported previously (Syers et al. 1969). Catchment soils have less Amox Fe and Amox Al and lower PRI than whole stream bank soil, whilst the <75-μm fraction of stream bank soils had more Amox Fe and Amox Al and higher PRI than catchment soils or whole stream bank soils. Stream bank soils, particularly the <75-μm fraction that can remain suspended in flowing streams and rivers, therefore have a significant potential to retain FRP. Whole stream bank soils and the <75-μm fraction are much less saturated with P than catchment soils. This is reflected in the very low PSR and Colwell P/PRI ratio of whole stream bank soils and the <75-μm fraction compared with catchment soils. The much lower Colwell P/PRI ratio for stream bank soils is due to their higher PRI and lower Colwell P compared with catchment soils. The lower PRI and higher PSR of catchment soils are due to continued direct exposure of these materials to the application of P-based fertilisers (Weaver and Reed 1998; Weaver and Wong 2011). Stream bank materials have limited direct exposure to the application of P-based fertiliser and are more likely to interact with P once it has been delivered to a stream. The low P content, high P sorption and low P saturation reinforces the notion that stream bank soils are more likely to retain P than contribute P as either PP or FRP. This is further reinforced by noting that the median TP content of whole stream bank soils in this study (94 mg P kg^−1^) was almost an order of magnitude lower than that reported by Laubel et al. (2003) (640 mg P kg^−1^) in a study where stream bank erosion contributed around half of the annual P load. Applying these findings to the potential for different soil materials to contribute to FRP (Moody 2011) or TP (Dougherty et al. 2011b) shows that >95 and >75 % of catchment soils will exceed the FRP (0.04 mg P L^−1^) target and TP (0.07 mg P L^−1^) water quality targets (ANZECC and ARMCANZ 2000), respectively. In contrast, <10 % of stream bank soils would exceed the TP target and around 10 % of stream bank soils would exceed the FRP target. These findings are commensurate with those of Agudelo et al. (2011) who showed that catchment soils could maintain much higher equilibrium P concentrations than deposited stream sediment or stream bank soils and that there was a potential that FRP released from deposited stream sediment could be adsorbed by stream bank materials. The low OC of whole stream bank soils and the <75-μm fraction compared to catchment soils is further evidence of their low fertility and suggests that they are largely mineral in origin. The broad chemical differences between these materials suggest that stream bank soils are exposed subsoil layers of common duplex (sand over clay) soils of the region that are depleted of P (McDowell et al. 2004). Stream bank soils are therefore more likely to retain P than contribute P directly as FRP or PP. This is supported by large-scale modeling for the south-west region of WA (NLWRA 2001) where it was reported that 14 % of the P could be sourced from stream banks as PP. Catchment soils could contribute either PP or FRP; however, runoff plot data suggests that catchment soils are mainly a contributor of FRP.

### Implications for the use of riparian buffers to manage P in catchments with sandy soils

**Fig. 7:**
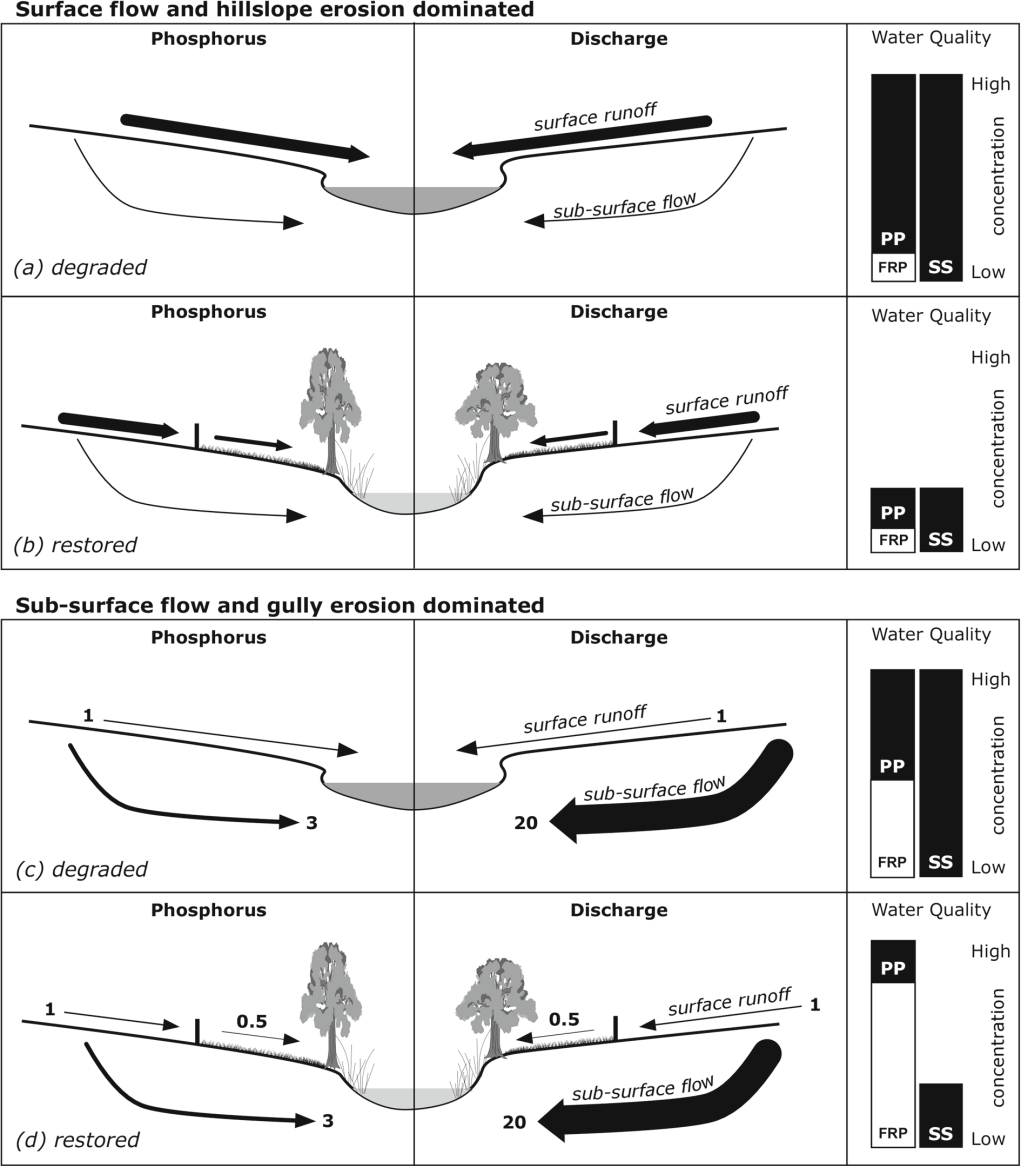
Conceptual models of P transport and transformations before (**a**, **c**) and after (**b**, **d**) riparian management for systems dominated by surface transport processes (**a**, **b**) and subsurface transport processes (**c**, **d**). Width of arrows and number annotations indicate amounts of discharge or P transported via each pathway relative to surface runoff. Adapted from data presented by McKergow et al. (2003, 2006a, b)

A previous riparian study in south-west WA (McKergow et al. 2003) showed that after improved riparian management (i.e. fencing, stock exclusion and revegetation), catchment SS exports fell from a mean of 150 kg ha^−1^ year^−1^ to less than 10 kg ha^−1^ year^−1^ due to reduced stream bank erosion. Whilst riparian buffers reduced total N (TN) by 25 %, they had no impact on TP and contributed to a P form change, where the median FRP increased by 70 % as particulate P had been swapped for FRP (Stevens and Quinton 2008). Experiments of riparian hydrology and water quality in south-west WA (McKergow et al. 2006a, b) showed that the surface trapping efficiency of nutrients and sediment was consistent with other published data that measured surface trapping efficiency (Sharpley et al. 2002). Grass buffers trapped 53 % of surface runoff, 54 % of TP, 50 % of FRP, 64 % of SS and 58 % of TN, whilst *Eucalyptus globulus* buffers trapped −3 % of surface runoff, 37 % of TP, 11 % of FRP, 21 % of SS and 42 % of TN. This compares well with the summarised data presented by Gitau et al. (2001) of TP removal by trees of 15 % (range 5–50 %) and 50 % (range 40–70 %) by grass filter strips. The work of McKergow et al. (2006a, b) also included subsurface flow and water quality measurements and showed 20 times more flow and 3 times more P discharged in subsurface flow than surface runoff (Fig. 7). Whilst 54 % of the surface-derived TP was trapped by grass buffers, this is discounted to around 10 % when both transport vectors are considered. For each unit of P transported over the soil surface and reduced by 50 % (or 37 % for trees), further 3 units of P transported by subsurface pathways are not attenuated. These measurements reinforce the hillslope P transport (“Hillslope P transport” of the “Results and discussion” section) and catchment P transport (“Catchment P transport” of the “Results and discussion” section) data, indicating that significant amounts of water and P travel via leaching and subsurface pathways. Additionally, the measurements are supported by NLWRA (2001) reports of only 3 % of P being sourced from hillslope PP in the region.

These previous riparian experiments can be interpreted in the context of the catchment (“Catchment P transport” of the “Results and discussion” section) and hillslope P transport (“Hillslope P transport” of the “Results and discussion” section) data and P characteristics of catchment and stream bank soils (“P characteristics of catchment and stream bank soils” of the “Results and discussion” section) to derive conceptual models of P management by riparian buffers in catchments with sandy soils. We hypothesise that prior to restoring riparian vegetation, FRP leached through the sandy soils and entered streams via subsurface pathways, and combined with available SS to give PP signals at the catchment outlet (Fig. 7c). Restoring riparian vegetation and stock exclusion stabilised stream banks and cut off SS supply, reducing the capacity for FRP to be adsorbed on to stream-derived particulates. Hence, whilst SS transport had been stopped, the more bioavailable FRP continued downstream (Fig. 7d). This contrasts with the most commonly understood model of riparian function where improvements in water quality are brought about through physically filtering and trapping hillslope-derived particulates containing P in surface runoff. Additionally, this alternative model is consistent with the hillslope P transport results (“Hillslope P transport” of the “Results and discussion” section) that also suggest a leaching and subsurface flow system dominated by FRP. These results are in striking contrast to the conventional conceptual model of P transport and riparian function provided in Fig. 7a, b. In the conventional model, P transported across the soil surface in particulate matter is the major contributor to P pollution (Fig. 7a). Restoration of the riparian system would trap the surface-derived P by physically filtering the particulate matter and associated P (Fig. 7b). In this environment, the management practice of riparian buffers does not match the dominant transport pathway or nutrient form.

Implications arise for catchments dominated by subsurface transport pathways in relation to swapping PP for FRP, as well as changes in the N/P ratio of discharging waters. If riparian buffers are implemented extensively and function similarly as reported here, discharged nutrients may cause ecosystem responses worse than already observed. For example, increased FRP may increase the frequency and intensity of algal blooms, and large-scale changes in the N/P ratio may force aquatic ecosystems to support undesirable algal species that can fix N from the atmosphere if N became limiting due to a reduction in catchment N exports of 25 %.

These results also bring into question the interpretation of large catchment-scale water quality measurements in terms of the implied mobilisation and transport processes from those measurements. In this case, PP was around 50 % of the TP (Fig. 7c) prior to implementing riparian buffers (McKergow et al. 2003). It could be assumed that surface runoff and erosion processes were responsible for the PP and, based on other published international research demonstrating the success of riparian buffers, that significant reductions in P transport would result. However, McKergow et al. (2003) showed that the removal of SS can lead to an increase in FRP. The runoff plot, catchment-scale water quality measurement and P retention and release characteristics of stream bank soils in this study point to a counter perspective—that the addition of SS could lead to a decrease in FRP and a proportional increase in PP in-stream.

Collectively, these studies demonstrate the importance of gaining an understanding of the prevailing hydrological and contaminant pathways within catchments, the nutrient retention and release characteristics of potential nutrient contributing materials, and nutrient transformations that may occur within stream systems prior to the wide-scale adoption of management practices. The results also imply that specific parts of catchments predisposed to surface transport processes and particulate nutrient transport would be the best candidates for riparian buffers if control of P was the aim. Ironically, retaining opportunities for SS in upstream P source areas of these catchments may assist to reduce downstream P transport. This would occur by adsorption of mobilised FRP to particulates that can then settle or be deposited onto floodplains by the longitudinal filtration of downstream intact riparian buffers. This is not an unreasonable assumption considering that NLWRA (2001) reports that only 39 % of mobilised P in this region is exported to downstream waterbodies, with the remainder retained in the hydrologic network.

## Conclusion

This paper takes data from a range of scales (in-stream potential for P retention, hillslope runoff plots, small and large catchment water quality monitoring) that could be used to infer an a priori case that riparian buffers are likely to have limited effectiveness in reducing P exports in catchments with sandy, low P sorption soils, where leaching of FRP is dominant. The findings help understand the processes behind previous reports of riparian ineffectiveness in this region. Before riparian buffers are recommended, investigations exploring riparian effectiveness, along with investigations that identify transport pathways and nutrient transformations, should be undertaken to help explain why riparian buffers may succeed or fail. This will assist to deliver a more cost-effective allocation of management effort and expenditure to nutrient management. Ignoring subsurface transport pathways could lead to a significant overestimation of the effectiveness of riparian buffers in this environment as large amounts of P appear to be transported as FRP via leaching and subsurface pathways. Hence, riparian buffers do not satisfy fit-for-purpose requirements for P management under the range of conditions presented here. Changes in P from PP to FRP as a result of riparian restoration and from FRP to PP as measurement scale moves from hillslopes to catchments could be explained by the removal or addition of P retentive stream bank soil. These dynamic changes in water quality measurements bring into question the interpretation of water quality data as representing the processes by which P is mobilised and transported.
